# Synergistic Effect of Microbial-Induced Carbonate Precipitation Modified with Hydroxypropyl Methylcellulose on Improving Loess Disintegration and Seepage Resistance

**DOI:** 10.3390/polym17040548

**Published:** 2025-02-19

**Authors:** Xingyu Wang, Hong Sun

**Affiliations:** State Key Laboratory of Ocean Engineering, School of Ocean and Civil Engineering, Shanghai Jiao Tong University, Shanghai 200240, China; wangxingyu@sjtu.edu.cn

**Keywords:** loess, microbial-induced carbonate precipitation (MICP), hydroxypropyl methylcellulose (HPMC), disintegration, seepage

## Abstract

Microbial-induced carbonate precipitation (MICP) is an eco-friendly soil stabilization technique. This study explores the synergistic effects of incorporating hydroxypropyl methylcellulose (HPMC) into the MICP process to enhance the disintegration and seepage resistance of loess. A series of disintegration, seepage, scanning electron microscopy (SEM), and mercury intrusion porosimetry (MIP) tests were conducted. The results show that HPMC forms protective membranes around calcium carbonate crystals produced by MICP and soil aggregates, which enhance cementation, reduce soluble salt dissolution, promote soil particle aggregation, and seal pore structures. At the optimal 0.4% HPMC dosage, the maximum accumulative disintegration percentage and the disintegration velocity decreased to zero. Additionally, HPMC-modified MICP reduced the amount, size, and flow velocity of seepage channels in loess. The integration of MICP with HPMC provides an efficient and sustainable solution for mitigating loess disintegration and seepage issues.

## 1. Introduction

Loess is a Quaternary sediment formed in arid and semi-arid climates. Due to the distinctive geological features of porous metastable structures and the susceptibility to water, loess is prone to disintegration upon contact with water, resulting in frequent geological disasters such as soil erosion, landslides, collapses, and mudslides [1]. Approximately 6.6% of China’s landmass is covered by loess, extending over 640,000 square kilometers. The largest and best-preserved loess layer on Earth is the Loess Plateau, covering an area of more than 380,000 square kilometers [2]. Survey statistics indicate that almost 30% of geological catastrophes occur annually in the Loess Plateau. In 2023 alone, the soil erosion area in the Loess Plateau reached 198,700 square kilometers, accounting for 52.29% of the total area. Enhancing the disintegration resistance of loess is significant for environmental protection, disaster control, and engineering safety in loess areas [3].

Typical intact loess is characterized by high porosity, open structures, and water-sensitive cementation between soil particles [4,5]. Cementation in a dry state has considerable strength in resisting deformation caused by external loads. However, the high porosity structure of loess quickly collapses when exposed to water [6]. Through computed tomography (CT) tests, Zhang et al. [7] found that the pore space of loess forms an intricate network with the intersection of pipelines and pores. Various factors, such as elevated water temperatures, decreased pressure during compaction, and lower initial moisture levels, facilitate the disintegration of loess [8,9]. Conventional soil improvement methods primarily employ lime, cement, fly ash, or epoxy resin to address the disintegration of loess [10,11,12,13,14]. However, these materials come with drawbacks, including excessive energy consumption, carbon emissions, high costs, and irreversible damage to the surrounding ecosystem. Therefore, researchers and engineers have been investigating more eco-friendly, energy-efficient, and sustainable methods for improving loess.

Microbial-induced carbonate precipitation (MICP) has been widely applied as a sustainable and environmentally friendly soil improvement technology. MICP utilizes urease produced by specific microorganisms, such as *Sporosarcina pasteurii*, to rapidly degrade urea into NH_4_^+^ and CO_3_^2−^ ions. These CO_3_^2−^ ions then chemically bond with Ca^2+^ on the surface of bacteria or in the solution across the cell membranes to create CaCO_3_ with cementing properties. Compared with traditional grouting materials such as cement mortar or epoxy resin, the bacterial suspension (BS) and cementing solution (CS) composed of urea and soluble calcium salts in MICP show higher permeability, allowing for deeper processing. At present, MICP is primarily used to enhance coarse-grained soils such as sand or gravel [15,16,17,18]. However, the treatment effect of MICP on fine-grained soils is limited [19,20,21,22]. In general, the repeated injection of BS and CS is necessary to achieve adequate strength and stiffness at a high cost of time and money. Atashgahi et al. [4] found that the amount of CaCO_3_ between loess particles increased with higher bacterial concentrations and longer curing times. Cheng et al. [23] demonstrated that applying 1 M CS after five cycles of MICP treatment effectively reduced soil erosion in loess. Chen et al. [24] investigated the effect of cement content and curing days on the unconfined compressive strength (UCS) of loess. They discovered that the UCS of loess treated with 1 M CS improved significantly after 14 days. Currently, there are limited studies using MICP for loess disintegration and seepage resistance improvement. Further research on MICP is crucial to expand the application of microbial geotechnology in fine-grained soils.

Recently, some additives such as solid fibers and polyacrylic amide (PAM) have been introduced into the MICP process to enhance the reinforcement effect of loess [22,25]. However, fibrous additives exhibit poor dispersion homogeneity and weak particle bonding, while monomers of PAM raise environmental toxicity concerns. In contrast, hydroxypropyl methylcellulose (HPMC), a naturally adhesive polymer, serves as a low-cost, renewable, and nontoxic soil stabilizer [26,27]. Ren et al. [28] evaluated the effects of different HPMC viscosities on soil water infiltration characteristics and conservative solute migration characteristics. The results showed that the cumulative infiltration amount, wetting front migration distance, and infiltration rate decreased significantly with increased HPMC viscosity, indicating that HPMC is a potential soil improver with the ability to hold water. Wu and Ren [29] investigated the effects of adding HPMC as a soil improver on soil water movement and water-stable aggregates. They found that HPMC reduced the infiltration and migration rates of conservative solute ions, which could enhance water and soil conservation while minimizing nutrient loss. Therefore, HPMC is a potential additive for MICP to further improve the bonding strength and seepage resistance of soil. Currently, there are very few applications of MICP combined with HPMC for soil improvement. Zhu et al. [30] used MICP and HPMC to improve the surface layer of sand. They found that the addition of HPMC can effectively improve the ammonia absorption rate and is effective in increasing the calcium carbonate content in the surface crust. The application of MICP combined with HPMC for loess improvement could provide new insights for developing more sustainable soil improvement technologies.

In this study, HPMC was introduced into the MICP process to consolidate loess. MICP-treated and HPMC-modified MICP-treated specimens were compared to demonstrate the improvement of loess disintegration and seepage resistance after HPMC addition. The mechanism by which HPMC-modified MICP enhances loess at the macroscopic and microscopic scales was analyzed in detail, considering the changes in the water environment and flow characteristics that were previously overlooked. This research aims to provide valuable insights into enhancing the engineering characteristics of loess, as well as the application of MICP in the field of fine-grained soils.

## 2. Materials and Methods

### 2.1. Loess and HPMC

The loess was obtained from Linfen City, located in Shanxi (SX) Province, China, at a depth of 5 m. Deposited during the upper Pleistocene of the Quaternary period (Q3), this loess exhibits a porous and loose structure, making it prone to collapse when submerged. The physical and mechanical parameters are listed in Table 1.

**Figure 1:**
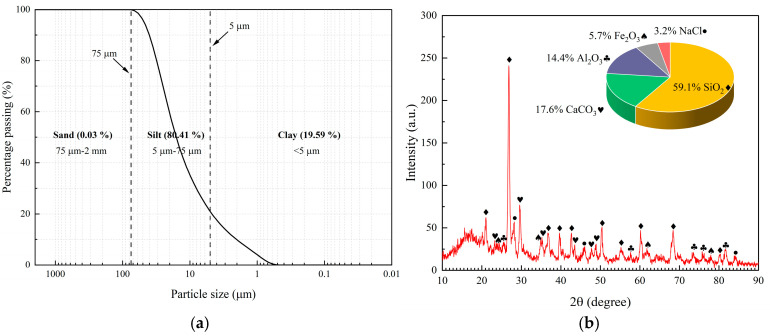

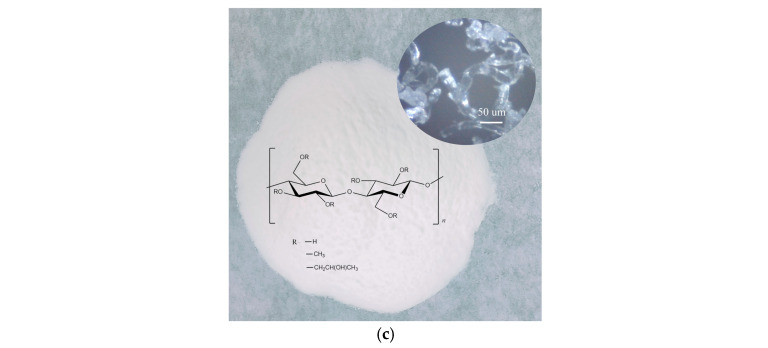
Composition of SX loess and HPMC: (**a**) grain size distribution of SX loess; (**b**) mineral composition of SX loess; (**c**) appearance and chemical composition of HPMC.

The grain size distribution of SX loess was determined using a S3500 laser particle size analyzer produced by Microtrac in the United States. Figure 1a shows that the SX loess consists of 80.41% silt, 19.59% clay, and 0.03% sand, which can be classified as low-plasticity clay (CL) according to the unified soil classification system (USCS) [31]. The mineral composition of SX loess was measured using X-ray diffraction (XRD) tests, as shown in Figure 1b.

HPMC was purchased from Tianjin Kemeiou Chemical Agent Co., Ltd. (Tianjin, China). It is a white powder with a density of 0.5 g/cm^3^, viscosity of 1 × 10^6^ MPa·s, and surface tension of 45 dyn/cm. The molecular formula and microstructure of HPMC are shown in Figure 1c. HPMC is easily soluble in cold water and has exceptional adhesive and film-forming characteristics. The optical micrograph shows that the microstructure of HPMC is characterized by cross-linked gel networks.

### 2.2. Preparation of BS and CS

**Figure 2:**
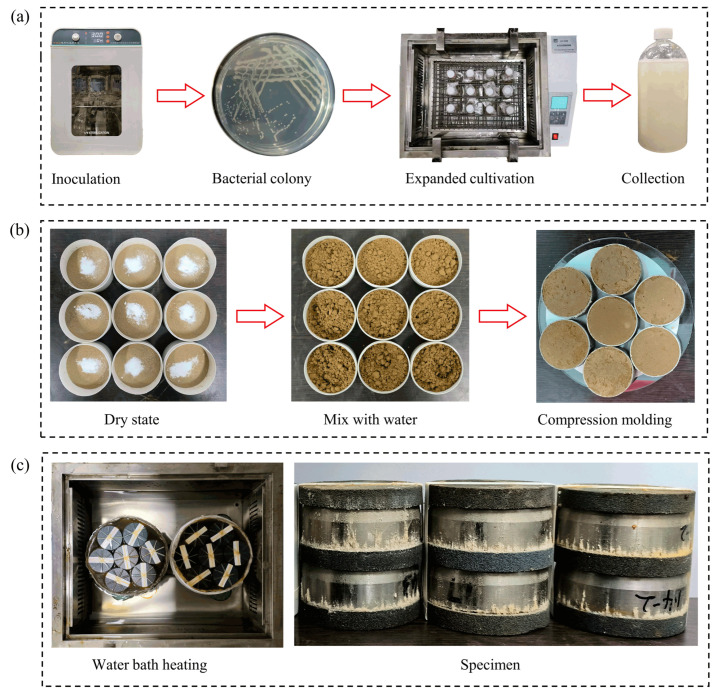
Microbial cultivation and specimen preparation: (**a**) Microbial inoculation and expansion cultivation; (**b**) Mixing and compression molding; (**c**) MICP treatment of specimens.

*Sporosarcina pasteurii* (ATCC 11859), purchased from China Shanghai Conservation Biotechnology Center (SHBCC) (Shanghai, China), was selected for MICP treatment. In order to obtain active BS, expanded cultures of the bacteria were carried out in a shaking table at 30 °C and 150 rpm for 48 h (Figure 2a). Each liter of liquid medium contains 5 g yeast extract, 15 g tryptone, 10 g NaCl, and 20 g urea. The BS used exhibited an optical density (OD_600_) of 1.5 ± 0.1. All media and glassware were sterilized at 121 °C and 1 MPa and then cooled to room temperature before use. The CS was formulated with 1 M urea and 1 M CaCl_2_.

### 2.3. Specimen Preparation

The collected SX loess sample was air dried, crushed, and passed through a 2 mm sieve. Then, the sieved loess was thoroughly dried in an oven at 105 °C for 24 h and cooled to room temperature. HPMC was mixed with dry loess in its dry state, after which water was added for the preparation of specimens (Figure 2b). The mass ratios of HPMC to dry loess were 0%, 0.1%, 0.2%, 0.4%, and 0.6%, respectively. During the mixing process, water was added to achieve a moisture content of 15% in the loess, ensuring the production of homogeneous specimens. Cylindrical specimens were used to avoid possible edge effects, and the mixed loess was carefully pressed through a jack into the ring cutter for compression molding at a constant dry density of 1.5 g/cm^3^. The ring cutter used for the disintegration test has a diameter of 61.8 mm and a height of 40 mm, while the specimen in the seepage test is 20 mm in height. Therefore, the volume of a cylindrical specimen for disintegration tests is 120 cm^3^, requiring 180 g of dry loess. To prepare a specimen with 0.2% HPMC content, 0.36 g of HPMC is needed. The required amount of HPMC for other contents follows accordingly. The disintegration tests and seepage tests for each HPMC content were conducted in triplicate.

The MICP treatment was conducted using the immersion method as follow: First, the ring knife containing loess was held tightly at the top and bottom by two pieces of permeable stones to prevent the specimen from swelling. Additionally, a piece of filter paper was placed at the interface between the specimen and the permeable stone to control soil particle loss. Two specimens and three permeable stones were tied together with rubber bands, as seen in Figure 2c. Next, the specimens were immersed in a stainless steel vessel containing successively injected BS and CS and heated in a water bath at 30 °C for 6 h. Evident calcium carbonate precipitation can be observed on the surface of the specimen in Figure 2c. Then, the specimens were cured at 30 °C and 98% humidity for 24 h. Finally, all treated specimens were dried at 60 °C to a constant weight and sealed with plastic wrap, ensuring an initial moisture content of 0%.

### 2.4. Disintegration Test

**Figure 3:**
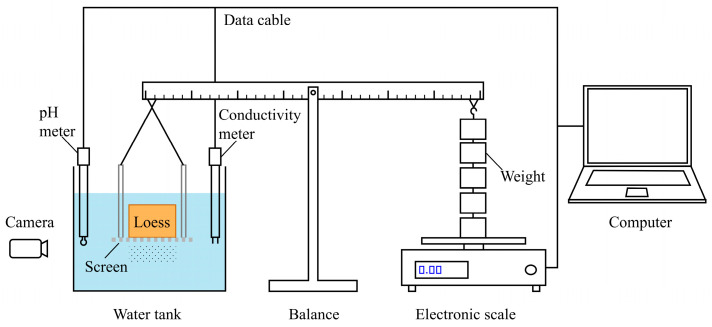
Schematic diagram of the disintegration test setup and data acquisition system.

Previous water immersion tests could only record the mass loss during disintegration. In this study, a new setup is designed to monitor the changes in the pH and EC of the solution in real time while recording the weight loss of loess, as shown in Figure 3. It consists of a water tank (200 mm × 140 mm × 160 mm), a screen, a balance, an electronic scale, a pH meter, and an electrical conductivity (EC) meter. The screen is a perforated steel plate with dimensions of 100 mm × 100 mm × 2 mm. It is uniformly distributed with circular apertures that are 5 mm in diameter and spaced 3 mm apart. The screen holding the loess specimen and the weights were hung at opposite ends of the balance. The weight on the right side is slightly greater than the left, giving the electronic scale an initial reading. During the disintegration process, the measured mass *m*, recorded by the electronic scale, will increase as a result of the mass loss from the specimen. At a temperature of 25 °C, the tank is filled with deionized water until the specimen is fully immersed. The volume of water injection for a single test is 2 L. The disintegration process is recorded by a camera, and the measured mass *m*, pH, and EC were captured at 1 s intervals by the data acquisition terminal.

### 2.5. Seepage Test

**Figure 4:**
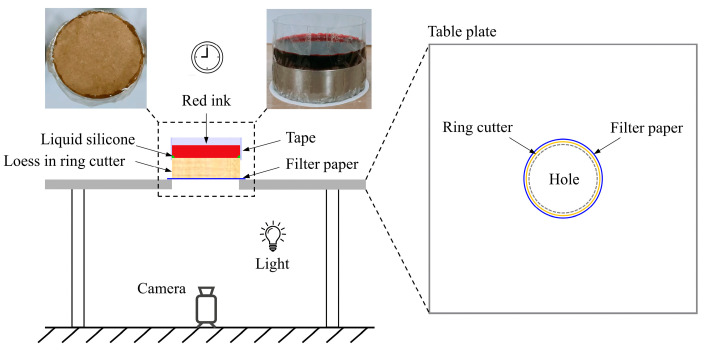
Schematic diagram of the seepage test setup.

Figure 4 illustrates a newly designed seepage test to observe the infiltration process of loess based on the strong adsorption capacity of CaCO_3_. Red ink is used as a color marker, and a ring of transparent tape is used to prolong the top surface of the specimen for ink retention. In addition, the bottom of the specimen is covered by a white filter paper. In order to prevent the direct leakage of ink along the inner wall, liquid silicone gel is applied to seal the contact between the top surface and the ring knife. Before the test begins, the specimen is positioned on a tabletop with a circular aperture, which is smaller than the ring cutter. Then, a clock is activated as soon as 50 mL of red ink is poured over the specimen. The wetting process of the filter paper is recorded by the camera placed under the table.

### 2.6. Scanning Electron Microscopy (SEM) Test

Soil samples approximately 1 cm^2^ in size at the center of the specimens were analyzed using the MIRA3 high-resolution field emission scanning electron microscope produced by TESCAN Corporation in the United States, with magnifications of 500×, 2000×, and 5000×.

### 2.7. Mercury Intrusion Porosimetry (MIP) Test

The MIP test serves as an effective technique for determining the pore size distribution characteristics of soil. The fundamental principle hinges on the concept that a non-wetting liquid, such as mercury, can penetrate into pores only when subjected to a specific pressure. The volume of liquid entering these pores increases with greater applied external pressure. The relationship between pressure and pore diameter, according to Washburn’s equation (1921), is given by

(1) p=−4Tcosθ/d

Here, *p* represents the intrusion pressure of mercury, *d* is the pore diameter, *θ* denotes the contact angle between mercury and soil, and *T* is the surface tension of mercury. The MIP tests utilized the AutoPore automatic mercury injection pore size analyzer, manufactured by the American Micromeritics Company, with related parameters T = 485.0 dyn/cm and *θ* = 140°. This instrument is capable of detecting pores within a size range of 0.003 to 1100 μm. To minimize boundary effects, the central portion of each soil specimen was selected for analysis. The chosen soil samples were first frozen with liquid nitrogen and subsequently dried via vacuum sublimation to preserve their microstructural integrity before undergoing MIP quantitative analysis. In order to reduce the errors associated with sample selection, 3 to 5 replicate samples subjected to the same consolidation pressure were analyzed using MIP tests. Representative results from these replicates were then selected for final quantitative analysis.

## 3. Results and Analysis

### 3.1. Analysis of the Disintegration Process

**Figure 5:**
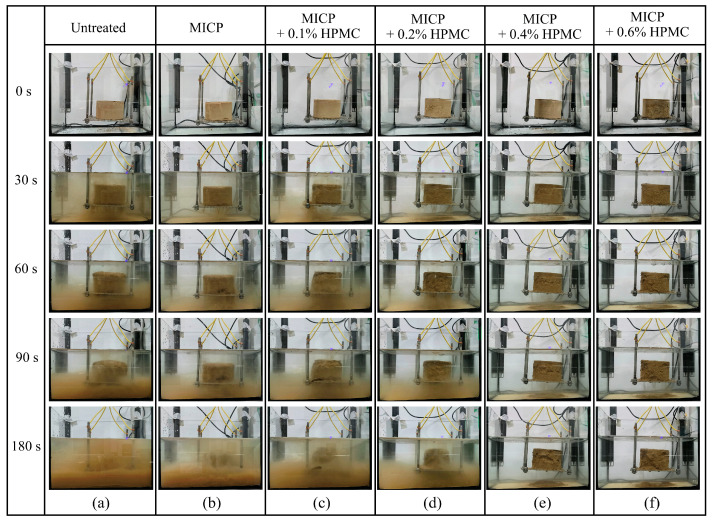
Photographs of loess disintegration process within the first 3 min: (**a**–**f**) Untreated, MICP-treated, MICP + 0.1%, 0.2%, 0.4%, and 0.6% HPMC-treated specimens, respectively.

Figure 5 shows the disintegration images of all loess specimens within the first 3 min. The untreated loess exhibited the most significant disintegration and volume expansion, accompanied by the highest water turbidity level. By contrast, Figure 5b demonstrates that the volume expansion and water turbidity of the specimen treated with MICP were reduced, suggesting that MICP can somewhat enhance the disintegration resistance of loess. Figure 5c–f show that HPMC can further prevent the disintegration of loess. With an HPMC content of exceeding 0.4%, the volume expansion of the specimens stopped, and the water began to become clear and transparent.

**Figure 6:**
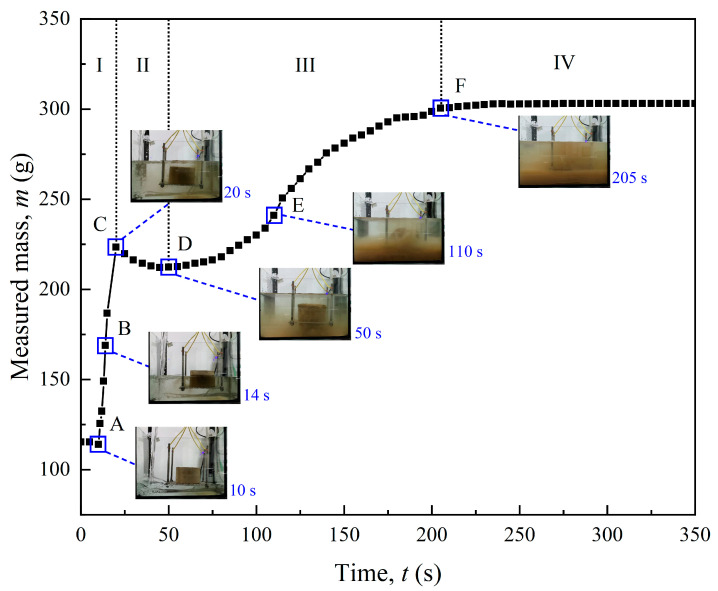
Disintegration process of untreated specimen.

Figure 6 shows the four stages of the disintegration process of untreated loess: water injection stage (I), water absorption stage (II), disintegration stage (III), and stabilization stage (IV). In stage I, as distilled water was injected into the tank at a constant rate, the mass *m* measured by the electronic scale rose rapidly over a short period due to the increasing buoyancy force on the specimen. When the whole specimen was immersed in water, *m* reached its first peak point, indicated at point C. Subsequently, water began to enter the inside and expel the air from the loess pores. In stage II, although a few loess particles separated from the matrix, *m* decreased steadily with time due to water absorption. Point D indicates that the specimen had significantly swollen with a slight water turbidity. From point D to F (stage III), the specimen experienced violent disintegration, with many soil particles and air bubbles spreading around. In stage IV, the screen showed almost no residue, indicating that the untreated loess specimen had completely disintegrated.

**Figure 7:**
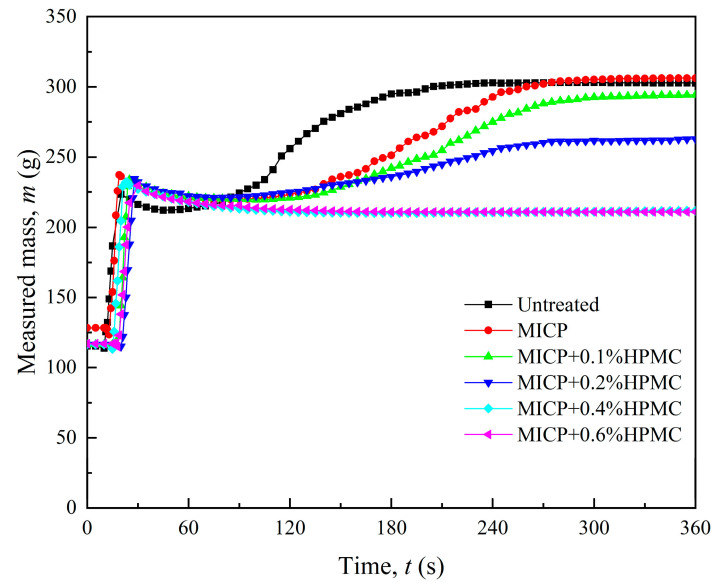
Measured mass versus time for different specimens in the disintegration tests.

The requirement to reinstall the specimen and reestablish the balance at the beginning of each disintegration test caused a slight fluctuation at point A in the disintegration curve, as shown in Figure 7. The disintegration curve of the loess treated with MICP exhibited a more gradual slope compared to the untreated loess. As the HPMC content increased, the period taken for disintegration increased. Notably, once the HPMC content was increased to 0.4%, the disintegration curves became approximately horizontal, demonstrating minimal disintegration during the first 6 min.

### 3.2. Disintegration Velocity

**Figure 8:**
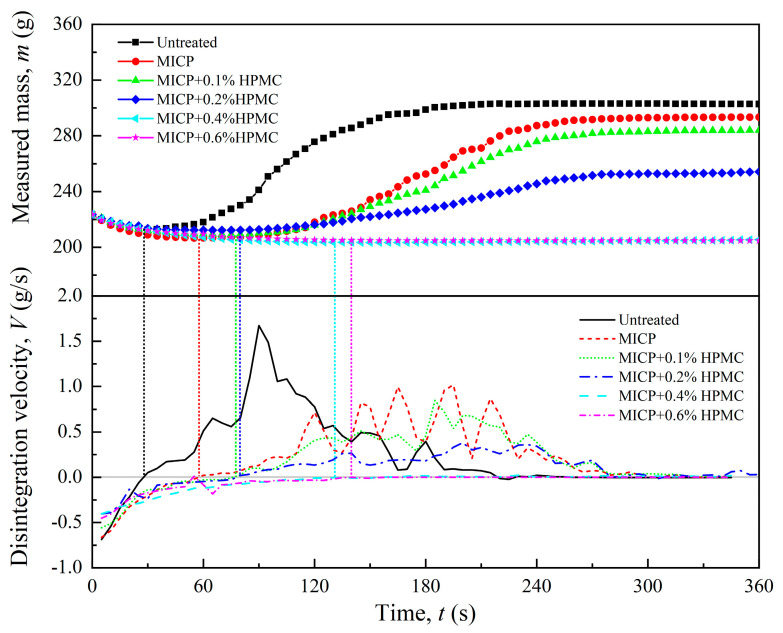
Data processing (the upper part) and disintegration velocity curves (the lower part).

All disintegration curves were aligned with the untreated loess disintegration curve at point C to start measuring time and weight, allowing for a comparison of the effects of HPMC-modified MICP on the degree and velocity of loess disintegration, as shown in Figure 8. In theory, the disintegration percentage and velocity should be calculated based on changes in the dry mass of the specimen. However, it is hard to measure the dry mass of specimens immersed in water accurately. In this study, the mass *m* measured by the electronic scale was used to calculate the disintegration percentage and velocity. Therefore, disintegration velocity curves can be obtained by calculating the time derivative of disintegration curves, as shown in the lower part of Figure 8. The untreated specimen has a negative disintegration velocity within the first 30 s, which corresponds to stage II in Figure 6. The continuous water erosion of the specimen resulted in the formation of many bubbles, thus quickly increasing the disintegration velocity (30–90 s). In the latter half of stage III (90–215 s), the disintegration velocity gradually declined to 0 g/s due to significant mass loss. Overall, the disintegration velocity curve of the untreated specimen exhibits a distinct unimodal trend, reaching a maximum velocity of 1.67 g/s.

The MICP-treated specimen displays a maximum disintegration velocity of 1.02 g/s, representing a 39% reduction compared to the untreated specimen. The disintegration velocity curve shows a multimodal characteristic. This is because the loosely arranged particles in loess were merged into multiple larger aggregates by calcium carbonate, leading to an uneven deceleration of the disintegration velocity. As the HPMC content increased to 0.2%, the maximum disintegration velocity of the specimen dropped to 0.38 g/s, a reduction of 77% from the untreated specimen. Furthermore, as the HPMC content rose to 0.4% and 0.6%, the disintegration velocity was stabilized at about 0 g/s in the first 6 min, showing significant disintegration resistance. Before disintegration occurs, loess undergoes a process of water absorption. Therefore, the point at which the disintegration velocity changes from negative to positive is defined as the disintegration starting point. Figure 8 shows a gradual delay in the starting point with the increase in the HPMC content, suggesting that the HPMC-modified MICP not only decreased the disintegration velocity but also prolonged the water absorption time.

### 3.3. Accumulative Disintegration Percentage

The measured mass *m* at any time in the DF segment and the measured mass *m*_D_ at point D are used for calculating the disintegration percentage (see Figure 6). The disintegration mass of the specimen is denoted as *m* − *m*_D_. If no soil matrix remains on the screen, the measured mass is expressed as *m*_0_. Therefore, the accumulative disintegration percentage *D* of loess is calculated as follows:

(2) $$D=(m-m_{D})/(m_{0}-m_{D})\times 100\%$$

**Figure 9:**
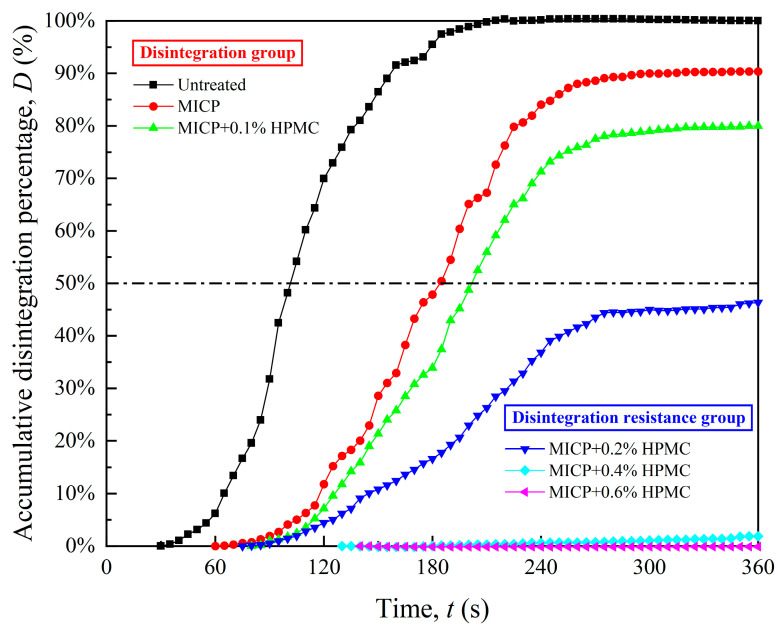
Accumulative percentage disintegration curves in the first 6 min.

Figure 9 depicts the accumulative disintegration percentage over time for different specimens during the first 6 min. The disintegration of untreated loess was complete, with the peak value *D*_max_ of the accumulative disintegration percentage curve reaching 100%. For the MICP-treated specimen, *D*_max_ decreased to 90.34%, which was still far from being acceptable in engineering practice. The addition of HPMC further reduced *D*_max,_ and the specimens were divided into two groups at a *D*_max_ of 50% in this study: the disintegration group (HPMC content less than 0.2%) and the disintegration resistance group (HPMC content not less than 0.2%). During the first 6 min, the disintegration group curves reached equilibrium, while the disintegration resistance group curves continued to show an upward trend. In addition, the disintegration resistance group shows a denser and smoother data distribution than the disintegration group.

**Figure 10:**
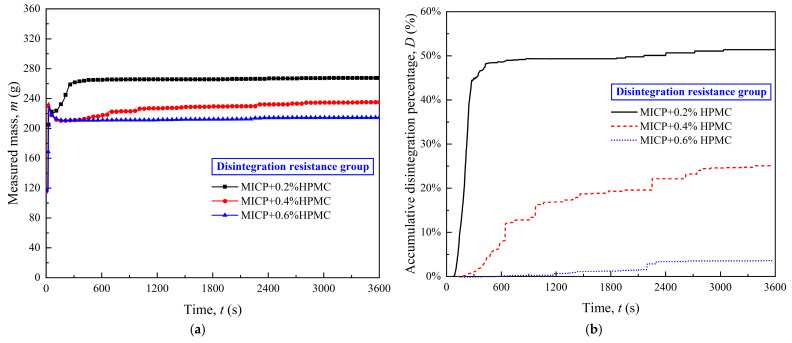
Disintegration process of the disintegration resistance group in 1 h: (**a**) measured mass change curves; (**b**) accumulative disintegration percentage curves.

Figure 10 depicts the disintegration and accumulative disintegration percentage curves of the disintegration resistance group within a one-hour period. A distinct stepwise evolution of the accumulative disintegration curves can be seen in Figure 10b. With the increasing HPMC content, the number of steps decreased, and the corresponding *D*_max_ values fell to 51.38%, 25.06%, and 3.62%.

**Figure 11:**
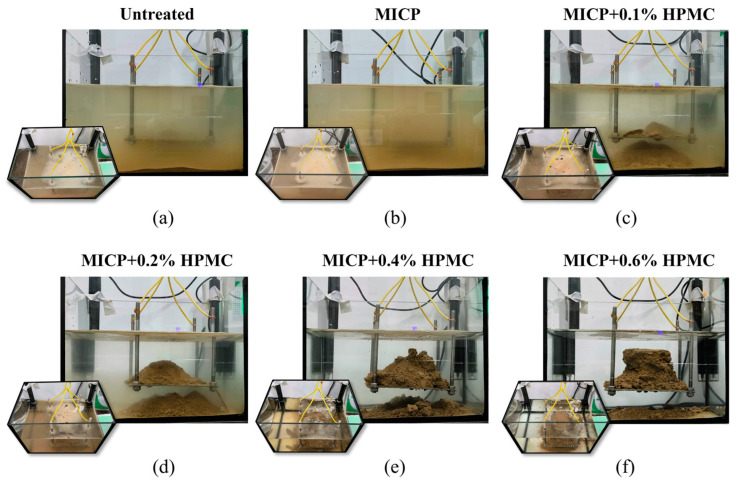
Photographs of disintegration degree and bubble distribution of different specimens after 1 h immersion in water: (**a**–**f**) Untreated, MICP-treated, MICP + 0.1%, 0.2%, 0.4%, and 0.6% HPMC-treated specimens, respectively.

Figure 11 displays the photographs of the remaining matrix and bubble distribution after the specimens were submerged in water for 1 h. In contrast to the clean and transparent water of the disintegration resistance group (Figure 11d–f), the water of the disintegration group (Figure 11a–c) is highly turbid with low visibility. The addition of HPMC facilitated the formation of a conical drift under the screen, and the size of the drift grew with the increasing HPMC content. At the same time, the integrity of the remaining matrix on the screen increased. Compared with the dense and stable bubbles in the disintegration group, the bubbles in the disintegration resistance group decreased significantly.

### 3.4. Water Absorption Capacity

**Figure 12:**
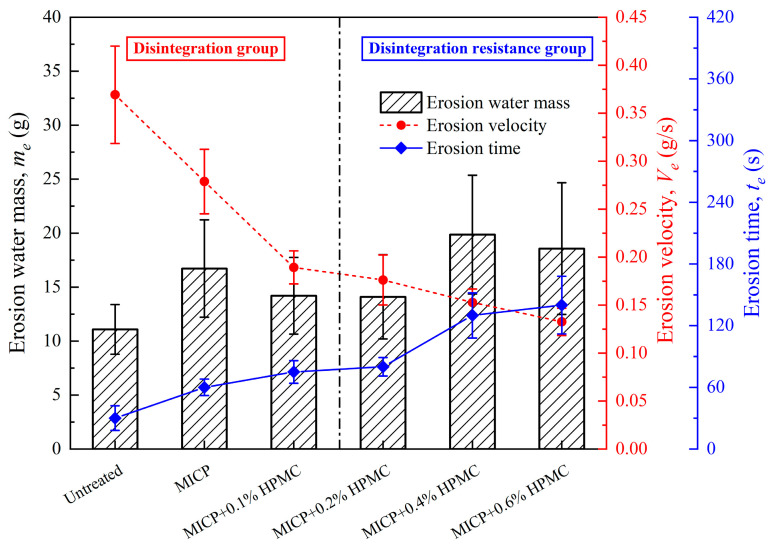
Erosion water mass, erosion velocity, and erosion time of different specimens at water absorption stage (II).

Figure 12 shows the water erosion mass, erosion velocity, and erosion time of different specimens in stage II. The untreated specimen exhibited the highest erosion velocity and the shortest erosion time. After the MICP treatment, calcium carbonate blocked certain pores in the loess, reducing the velocity of water erosion and extending the absorption time. It is worth noting that the water absorption mass of the MICP-treated specimen is higher than that of the untreated one. This is because the incomplete filling of calcium carbonate divided the large pores into multiple smaller ones, enhancing the capillary action and adsorption capacity of the loess. HPMC further sealed the tiny pores in the loess, causing the specimen treated with MICP + 0.1% HPMC to absorb less water than the MICP-treated specimen. It should be noted that water absorption (causing a decrease in the electronic scale reading) and disintegration (causing an increase in the electronic scale reading) occurred simultaneously, so the total water absorption calculated from the disintegration curve is underestimated, especially for the disintegration group. As the HPMC content increased to 0.2%, the specimens showed strong disintegration resistance, which further prolonged the water absorption time and reduced the underestimation effect. Therefore, the water erosion mass increased with the increasing HPMC content.

### 3.5. EC and pH

**Figure 13:**
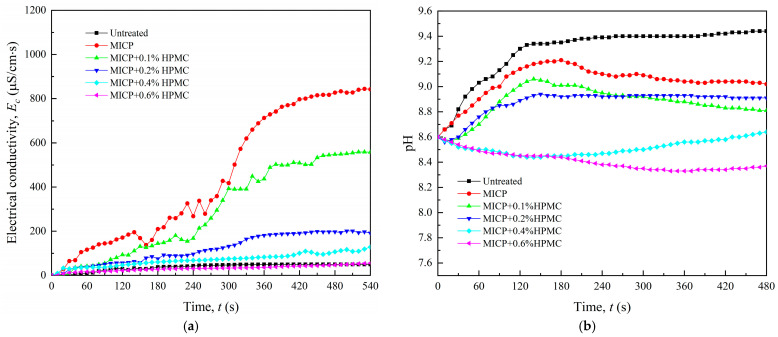
Variation in electrical conductivity (**a**) and pH (**b**) with time of different specimens.

The EC and pH of the solution will change with the dissolution of electrolytes in loess during the disintegration process. Figure 13 shows the variation in EC and pH over time for different specimens. The untreated specimen contained limited soluble ionic compounds, and the EC of the solution only rose from 0 μS/cm to 49.3 μS/cm (see Figure 13a). By contrast, the specimen treated with MICP has a maximum conductivity of 844 μS/cm. This is because both the dissolution of calcium chloride and the catalytic decomposition of urea in CS greatly increased the EC of the solution. Subsequently, the rising speed and maximum value of EC decreased with increasing HPMC content. On the one hand, the *D*_max_ of the specimen decreased with the increase in HPMC content, meaning that the soluble substances in loess decreased. On the other hand, HPMC created a polymer layer to seal off loess particles and aggregates, which helps to reduce mineral solubility.

All curves in Figure 13b show an increase in pH with increasing disintegration degree. The untreated specimen had the greatest pH, whereas the MICP treated specimen showed a slight reduction in pH. This is because the hydrolysis of calcium ions in CS and the hydrolysis of ammonium ions, a byproduct of urea decomposition in the MICP process, increased the concentration of hydrogen ions. The hydrolysis reaction equations are as follows:

(3) $${\mathrm{CO}(\mathrm{NH}}_{2}{)}_{2}+2H_{2}O\to {\mathrm{CO}}_{3}^{2-}{+\mathrm{NH}}_{4}^{+}$$

(4) $${\mathrm{Ca}}^{2+}{+2H}_{2}O⇌{\;\mathrm{Ca}(\mathrm{OH})}_{2}+2H^{+}$$

(5) $${{\mathrm{NH}}_{4}}^{+}{+H}_{2}O⇌{\;\mathrm{NH}}_{3}•H_{2}O+H^{+}$$

The pH of the solution continued to show a decreasing trend with increasing HPMC content. It can be elucidated that the decrease in *D*_max_ resulted in a corresponding reduction in alkaline leaching substances. It is worth noticing that the pH of the solution declined rather than increased as the HPMC content increased to 0.6%. As shown in Figure 11f, the specimen treated by MICP + 0.6% HPMC remained intact, indicating that the dissolution of alkaline substances in loess is negligible. Finally, the hydrolysis of calcium and ammonium ions caused a minor reduction in pH. Ren et al. [28] confirmed analogous results in their investigation into how HPMC influences the migration of solutes in soil, using chloride ions as tracers. Their findings indicate that HPMC can significantly slow down the transport of solutes. The cumulative infiltration amount, wetting front migration distance, and infiltration rate decreased significantly with increased HPMC amount. In practical engineering applications, the effectiveness of HPMC in reducing the solubility of soluble salts helps to minimize nutrient loss and enhances the stability of cementation between soil particles in loess.

### 3.6. Seepage Resistance Analysis

**Figure 14:**
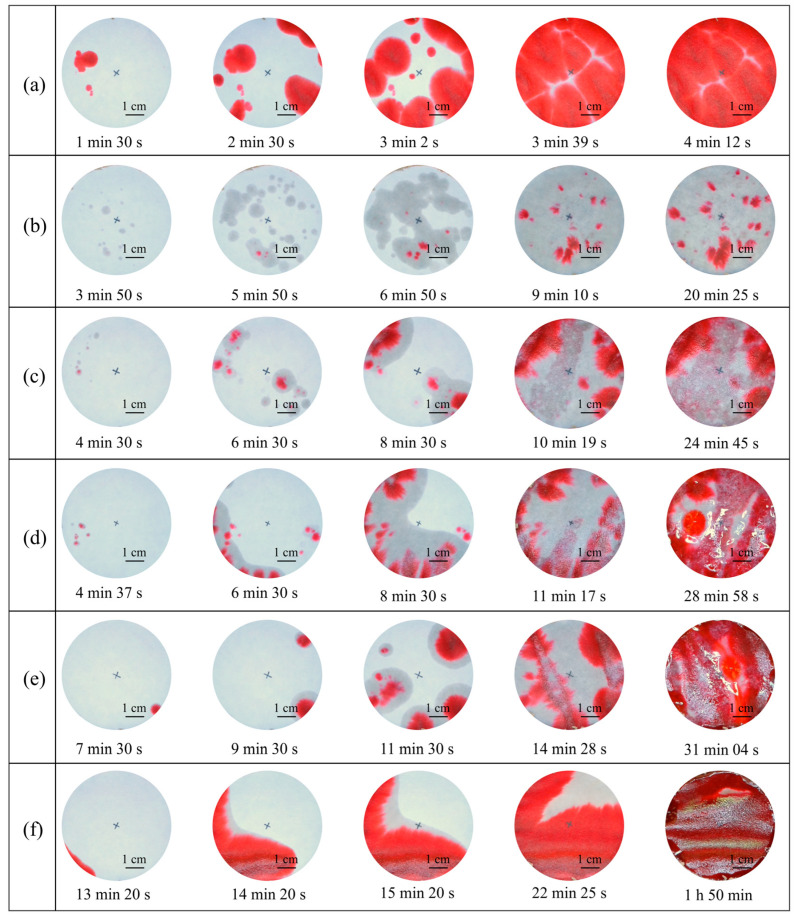
Changes in color and water distribution of filter papers under different specimens in the seepage test: (**a**–**f**) Untreated, MICP treated, MICP + 0.1%, 0.2%, 0.4% and 0.6% HPMC treated specimens, respectively.

**Figure 15:**
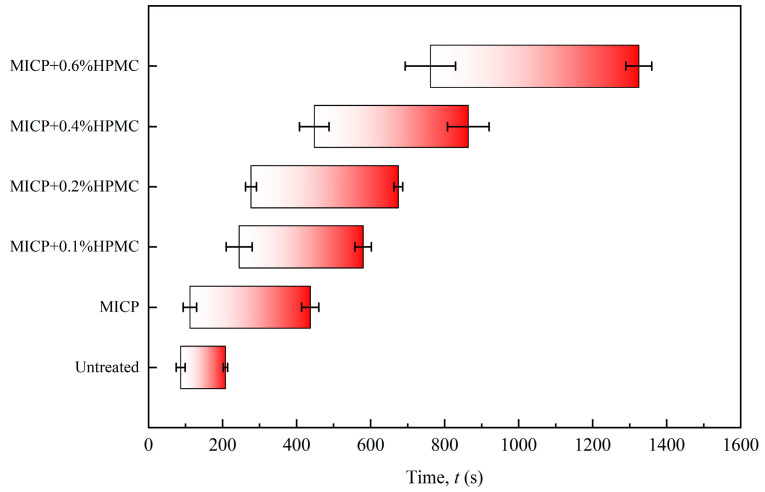
Starting time and total wetting duration of filter papers for specimens treated by different methods.

Figure 14 displays the photographs of filter papers in the seepage tests for different specimens. The first photo was taken when watermarks first appeared on the filter paper. The fourth photograph was taken when the filter paper was saturated with water, and the fifth photograph was taken after the 50 mL of ink had completely penetrated the loess. Figure 15 shows a bar chart of the duration from the first photo to the fourth photo. Notable increases are observed in the starting time of ink reaching filter paper and the total wetting duration. The first column of photos shows a trend of increasing and then decreasing watermarks on the filter paper. The same phenomenon is notable in the second column. As described in the water absorption capacity analysis, calcium carbonate fills and splits large pores into multiple smaller pores, thereby reducing flow velocity and increasing seepage channels. Hence, compared to Figure 14a, there are more watermarks in the same column of photos in Figure 14b. In addition, unlike the red ink dots in Figure 14a, the marks in Figure 14b have almost no color. This is attributed to the high adsorption capacity of CaCO_3_, which effectively separated and absorbed the pigments from the ink. As the HPMC content increased, more seepage channels were blocked. Thus, the spots reached the filter paper decreased again. Furthermore, the red area expanded with the increase in HPMC content because HPMC weakened the adsorption capacity of CaCO_3_.

**Figure 16:**
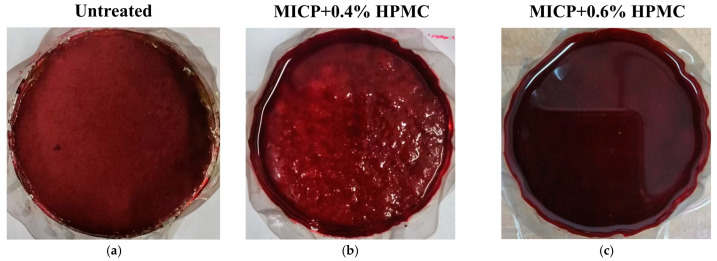
Ink accumulation on the top of specimens at the moment of exactly fully wetting of the filter paper: (**a**) Untreated; (**b**) MICP + 0.4% HPMC; (**c**) MICP + 0.6% HPMC.

For a given total volume of water, the more water absorbed by the specimen, the less water reaches the filter paper. The last column of photos in Figure 14 shows an increasing amount of water collected on the filter paper with increasing HPMC content, indicating a decrease in the absorption capacity of loess. Figure 16 shows the ink accumulation on the top surfaces of three specimens when the filter papers were just fully wetted. As seen in Figure 16a, the untreated specimen has nearly no liquid on the surface, meaning that all the ink has adequately penetrated. Due to the lack of ink source from the top surface, the fifth photograph in Figure 14a is essentially the same as the fourth. By contrast, the excess ink accumulation on the top surfaces in Figure 16b,c correspondingly produced visible droplets in the last column photos of Figure 14e,f.

### 3.7. Macro and Micro Structural Characteristics

**Figure 17:**
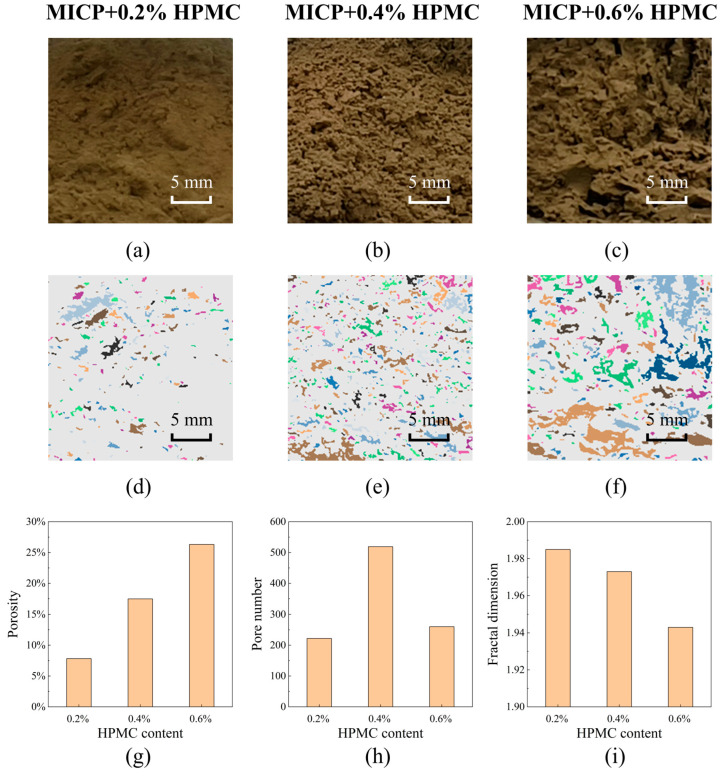
Macrostructural characterization of the remaining matrix surface for specimens in the disintegration resistance group (specimens treated by MICP + 0.2%, 0.4%, and 0.6% HPMC, respectively): (**a**–**c**) macrostructural photographs; (**d**–**f**) pore identification by binary processing; (**g**–**i**) porosity, pore number, and fractal dimension of pore size distribution.

The images in Figure 17a–c provide close-up views of the matrix remaining on the screen of the disintegration resistance group to characterize the effect of HPMC-modified MICP on the macroscopic structure of loess. The photos of the disintegration group were unavailable due to the limited amount of residual soil and poor water clarity. With an increase in the HPMC content, the soil aggregation degree increased. Numerous “pores” appeared on the surface of the matrix. In fact, the grain size of loess is so small that its primary pores are imperceptible to the camera. These “pores” are mainly left by the detachment of the soil blocks from the matrix. The detached blocks and the remaining matrix are in a complementary relationship, allowing for the binarization and zoning of the “pores” to quantify the degree of soil particle aggregation, as shown in Figure 17d–f. The porosity, pore number, and fractal dimension of pore size distribution are represented in Figure 17g–i. The porosity rises with the increasing HPMC content, indicating that HPMC is favorable for forming large aggregates. In theory, the number of detached blocks should decrease with the increasing aggregation degree. However, Figure 17h shows that the specimen treated with MICP + 0.2% HPMC displays the fewest pores. The reasonable explanation is that soil particles from elsewhere covered up the previous small pores created by the collapse of tiny aggregates. The connection of pores can be described by the fractal dimension of pore size distribution. A lower fractal dimension indicates a higher level of pore connection. The fractal dimension reduced with the rise in the HPMC content, indirectly reflecting the enhancement of the aggregation degree of loess.

**Figure 18:**
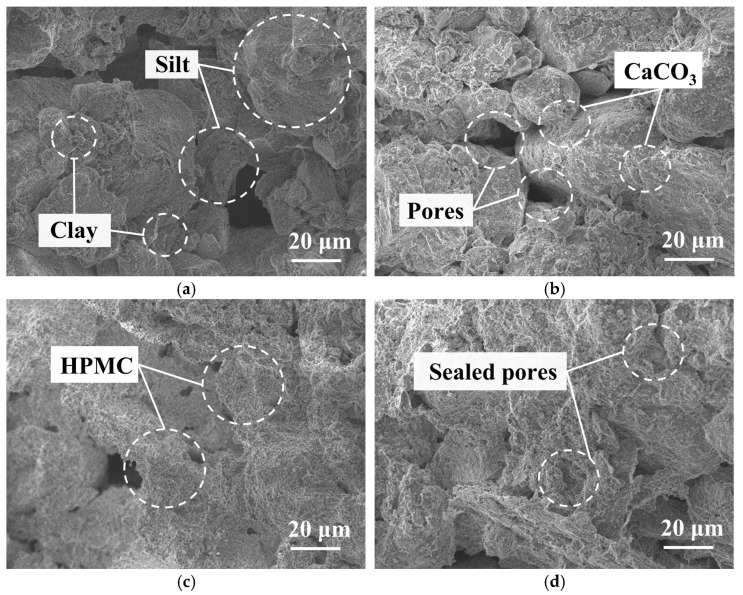
The SEM images of loess treated by different methods: (**a**) untreated loess; (**b**) MICP-treated loess; (**c**) MICP +0.4% HPMC-treated loess; (**d**) MICP +0.6% HPMC-treated loess.

Figure 18 shows the SEM images of specimens treated with different methods. The soil structure of untreated loess is composed of a large number of translucent elliptical silt particles, as depicted in Figure 18a. Some silt particles are cemented together by smaller clay particles, whereas others are loosely packed into metastable structures. These pores aid in material migration and structural rearrangement during water erosion, also acting as seepage channels. The clay particles bridged between silt particles contribute to the stability of the structure. However, when exposed to water, the clay particles may swell, leading to immediate collapse. Figure 18b demonstrates that the filling of calcium carbonate contributes to the compaction of the loess structure. For the HPMC-modified MICP-treated specimen, in addition to being filled with calcium carbonate, the membrane structures formed by HPMC can further seal the pores, as shown in Figure 18c,d.

### 3.8. Quantitative Microscopic Analysis

**Figure 19:**
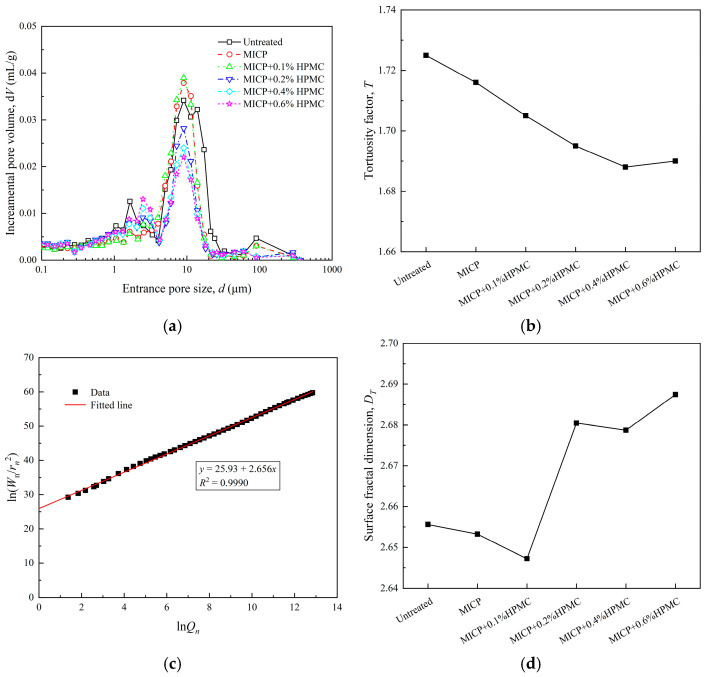
The quantitative microscopic results of loess treated by different methods from MIP tests: (**a**) Pore size distribution curves; (**b**) Tortuosity factor; (**c**) The relationship between the surface energy to the *n*th mercury intrusion *W_n_* and the *n*th mercury intrusion increment *Q_n_*; (**d**) surface fractal dimension.

Figure 19 presents the quantitative analysis results obtained from MIP tests to investigate the impact of varying HPMC contents on the microscopic pore structure of loess. Specifically, Figure 19a illustrates how changes in HPMC content affect pore size distribution (PSD). For untreated loess, pore diameters are predominantly distributed around 5–20 μm, consistent with observations from SEM images shown in Figure 18a. After MICP treatment, the PSD curve shifts leftward with an increase in peak height, indicating that the filling effect of calcium carbonate transforms larger pores (10–20 μm) into smaller ones (5–10 μm). As the HPMC content increases, more HPMC membrane structures progressively seal the smaller pores (5–10 μm), leading to a gradual decrease in the PSD peak within the 5–10 μm range. Meanwhile, there is a noticeable increase in the number of pores within the 1–5 μm range. Correspondingly, SEM images visually illustrate the evolution of pore structures, as shown in Figure 18c,d.

The tortuosity factor represents the average ratio between the actual length of the pore channels and the straight-line distance between the ends of those channels in the soil, which can reflect the complexity of the seepage channels. A higher tortuosity factor indicates more complex shapes and distributions of the seepage channels. Figure 19b shows that the tortuosity factor decreased as the HPMC content increased, and it gradually leveled off after the HPMC content reached 0.4%. This suggests that HPMC effectively sealed the pores in loess, significantly reducing the complexity of the seepage channels.

The impact of the HPMC membranes on the pore structure of loess can be quantitatively analyzed by calculating the surface fractal dimension from the MIP test results. A larger surface fractal dimension indicates a more complex surface structure within the loess. The thermal dynamics-based expression for the fractal dimension *D_T_* is as follows:

(6) $$\ln\left(W_{n}/r_{n}^{2}\right)=D_{T}\ln Q_{n}+C$$

where $W_{n}=\sum_{i=1}^{n}P_{i}\Delta V_{i}$, denoting the surface energy required for accumulation to the *n*th mercury intrusion; $Q_{n}=V_{n}^{1/3}/r_{n}$, denoting the *n*th mercury intrusion increment, *r_n_* represents the radius associated with the *n*th mercury intrusion increment; and C is a constant.

The linear relationship between ln(*W_n_*/*r*_n_^2^) and ln*Q_n_* is shown in Figure 19c, and the changes in *D_T_* caused by different HPMC contents are illustrated in Figure 19d. When the HPMC content is below 0.1%, *D_T_* shows a decreasing trend. This decrease is attributed to the reduction in pore space due to the filling and coating effects of calcium carbonate and a small amount of HPMC. After the HPMC content reached 0.2%, the surface fractal dimension suddenly increased and continued to rise with further increases in the HPMC content. This is because more HPMC membranes sealed the pores, bridged between soil particles, or stacked on the surface of soil particles, gradually forming a more complex surface structure, as shown in Figure 18c,d.

## 4. Discussion

### 4.1. Enhancement Mechanism of Loess Disintegration Resistance

Disintegration will occur only when the disintegration force surpasses the cohesive force of loess [32,33]. In loess, the disintegration force is determined by the wedging force of water film and the swelling force of wetted clay minerals. The cohesive force comes from the cementation of soil particles by soluble salts and clay minerals. Insufficient compaction and cementation in loess lead to the formation of numerous metastable structures, which are susceptible to sudden collapse in water. On the one hand, as a polar nonionic cellulose ether, HPMC has high solubility in water. On the other hand, the calcium carbonate clusters generated by microorganisms possess a high specific surface area, which facilitates their exceptional adsorption capabilities. Chen et al. [34] found that HPMC can form a colloid film with a 3D network structure in water and adsorb onto the surface of cement and sand particles, reducing the migration capability of water within cement mortar. Similarly, the polymer gel film produced by HPMC can firmly adhere to the surface of calcium carbonate. It functions as a reinforcing and waterproofing agent, thereby enhancing the cohesion and water stability of loess and decreasing the swelling force of the clay minerals. Figure 13 demonstrates that HPMC-modified MICP can lower the EC of water during disintegration, implying that the gel film created by HPMC and calcium carbonate envelops the clay particles well and alleviates the decrease in cement strength caused by the dissolution of soluble salts.

The disintegration of loess occurs from the outside inward, during which water infiltrates the soil and squeezes out the air in the pores, creating bubbles that escape outward. The faster the water invades, the more air is pushed out in a short time, leading to a dramatic rise in the quantity and volume of bubbles (Figure 11a). As the bubbles rise, their volume expands due to the decreasing surrounding pressure, causing soil particles to detach from the surface of the matrix and spread in all directions. Air compression by water generates repulsive forces, which may cause disintegration if they exceed the cementing forces between soil particles [35,36].

The membrane structure formed by HPMC combined with calcium carbonate effectively reduced the invasion velocity of water by plugging, isolating, and reinforcing the pores in loess (see Figure 12). As a result, the frequency and quantity of bubbles are significantly reduced (Figure 11d–f), mitigating the negative impact of bubble expansion on the loess structure. The reinforcement of cementation, reduction in soluble salt dissolution, increase in particle aggregation, and independent sealing of pore space collectively enhance the disintegration resistance of loess. Regarding the effect of MICP treatment on loess disintegration, Zhang et al. [22] discovered that MICP could only extend the disintegration time from 10 to 25 min. In contrast, loess treated with HPMC-modified MICP shows significantly improved stability, with most aggregates remaining on the screen even after 1 h. This indicates that HPMC-modified MICP is an effective approach to addressing loess disintegration issues.

### 4.2. Microscopic Mechanism of Loess Seepage Resistance

**Figure 20:**
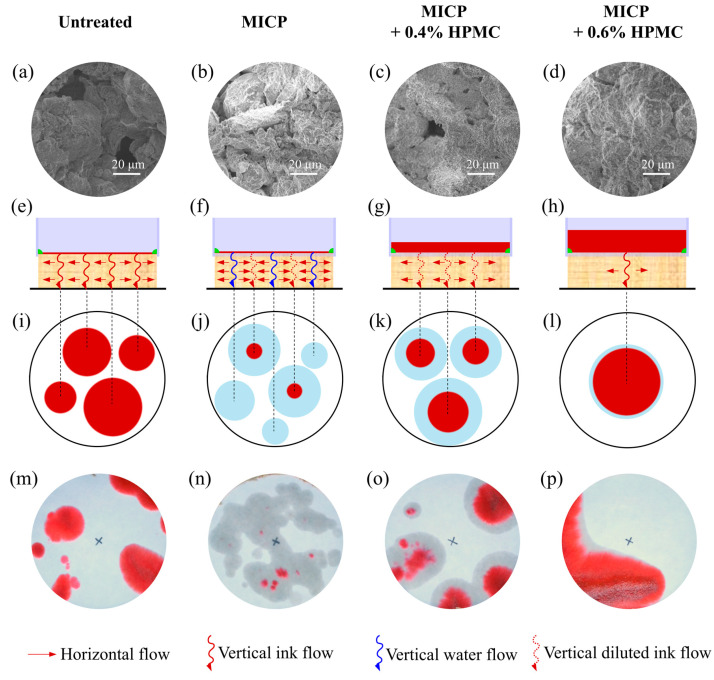
Seepage characteristics and micro-mechanisms of loess treated with HPMC-modified MICP: (**a**–**d**) typical SEM images of the local detail structures; (**e**–**h**) schematic diagram of flow characteristics and surface ink accumulation; (**i**–**l**) schematic diagram of water marks; (**m**–**p**) photographs of filter papers in seepage tests.

The microscopic mechanism of improved loess was analyzed based on the results of the seepage test and SEM images, as shown in Figure 20. Regarding the main flow directions, the seepage is classified into horizontal and vertical seepage, respectively. Figure 20a shows that the untreated loess contains many embedded pores with large diameters and low adsorption capacity. During the seepage process, the ink flowed rapidly throughout the untreated specimen without any separation of pigment separation (Figure 20e), resulting in the formation of uniformly distributed red circular patterns (Figure 20i). The soil particles and pores in loess treated with MICP are coated or filled with a significant quantity of calcium carbonate (Figure 20b). The ink was diluted or even fully decolorized due to the adsorption of pigments by the calcium carbonate (Figure 20f). With the filling and division of the large pores into smaller ones, the flow velocity of a single seepage dropped, but the number of seepage channels significantly rose. Correspondingly, many smaller colorless water marks appeared on the filter paper, as shown in Figure 20n. As a polymer material with excellent film-forming properties, HPMC can further seal the rest of the small pores, and the higher the content, the better the sealing effect. As a result, the number of seepage channels decreased with the increasing HPMC content. At the same time, more ink was isolated on the top surface of the specimen, as shown in Figure 20f–h. In addition, the adsorption capacity of calcium carbonate decreased by HPMC encapsulation. Eventually, the number of seepage channels declined while the ratio of the red area to the total area of a single spot increased, as depicted in Figure 20j–l.

The infiltration characteristics of loess can be evaluated in two ways. On a macroscopic level, it can be measured by the amount of seepage per unit time. On a microscopic level, it can be assessed by the number of seepage channels and the seepage velocity. As mentioned above, the gel-like compound formed by calcium carbonate and HPMC can block the pores in loess, reducing the number of seepage channels and sealing pore throats. This results in lower permeability and a longer penetration time. Even larger seepage channels can be narrowed by a layer of calcium carbonate gel formed by HPMC-modified MICP on the inner walls, which expands upon water absorption. Furthermore, the hydrophilic groups on HPMC molecules can establish hydrogen bonds with water, increasing the viscous resistance between the channel wall and seepage, thereby minimizing the seepage velocity. Ren et al. [28] investigated the effect of HPMC on the pore water flow velocity in silty soil. The experimental results showed that the average pore water flow velocity decreased by 13.6% to 28.2%, confirming the effectiveness of HPMC in reducing seepage flow velocity. Correspondingly, as illustrated in Figure 15, the time intervals for wetting the filter paper in the seepage tests were noticeably extended with the increase in the HPMC content.

To summarize, reducing the number of seepage channels, seepage diameter, and seepage velocity at the microscopic scale improves the seepage resistance of loess. It should be emphasized that the seepage and disintegration resistance of loess are interdependent [32]. Enhancing the seepage resistance results in a decrease in water erosion velocity, thereby reducing the wedging force of water film and the expansion force of bubbles, which helps improve the disintegration resistance of loess.

### 4.3. Limitations

While this study provides valuable insights into the application of MICP and HPMC for loess improvement, several limitations should be noted. First, the experimental conditions were controlled, which might not fully reflect real-world scenarios where environmental factors could vary significantly. Additionally, while it is relatively easy to achieve the uniform mixing of HPMC with small samples of loess, further exploration is needed to address how to ensure uniform mixing in large-scale engineering applications involving substantial volumes of loess. Lastly, practical engineering requires that treated loess maintains long-term stability. However, this study did not investigate the long-term effects of MICP and HPMC treatments under various environmental conditions.

Future research should focus on the long-term stability and process optimization of large-scale, HPMC-modified, MICP-treated loess under complex environmental conditions. This will help facilitate the practical application of this technology for loess improvement in engineering projects.

## 5. Conclusions

This paper investigates the effect of HPMC-modified MICP on the disintegration and seepage resistance of loess. Based on a series of disintegration, seepage, SEM, and MIP tests, the enhancement mechanisms of HPMC-modified MICP on the disintegration and seepage resistance of loess were analyzed at the macroscopic and microscopic scales. The following conclusions can be drawn:

(1) MICP can enhance the structural stability of loess by filling pores and cementing soil particles with calcium carbonate, although its effectiveness in improving the disintegration resistance of loess is limited. The maximum accumulative disintegration percentage decreased from 100% to 90.34%, and the maximum disintegration velocity dropped from 1.67 g/s to 1.02 g/s. The formation of aggregates altered the shape of the disintegration rate curve from a single peak to multiple peaks.
(2) MICP and HPMC synergistically enhance the disintegration resistance of loess. HPMC is able to form membranes wrapping around soil aggregates and calcium carbonate to further enhance loess structure. With the increasing HPMC content, both disintegration velocity and frequency decreased, and little disintegration occurred after the HPMC content increased to 0.4%. The stronger calcium carbonate cementation, reduced soluble salt dissolution, increased particle aggregation, and improved sealing of pores collectively enhanced the disintegration resistance of loess.
(3) HPMC can further seal the small pores left unfilled by calcium carbonate, enhancing the seepage resistance of loess by reducing the amount, size, and flow velocity of seepage channels. As an environmentally friendly and efficient soil treatment technology, HPMC-modified MICP provides a new solution to address the disintegration and seepage resistance issues of loess.

## Figures and Tables

**Table 1:** Physical and mechanical properties of SX loess.

Specific Gravity *G*s	Liquid Limit *w*_L_ (%)	Plastic Limit *w*_P_ (%)	Plasticity Index *I*_p_	Dry Density *ρ*_d_ (g/cm^3^)	Natural Moisture Content *w* (%)	Air Dry Water Content *w*_a_ (%)
2.69	27.75	16.61	11.14	1.35	9.13	1.18

## Data Availability

The original contributions presented in this study are included in the article. Further inquiries can be directed to the corresponding author.
